# Supplementary figures and images for: Multivariable G-E interplay in the prediction of educational achievement
Source: PLoS Genet. 2020 Nov 17;16(11):e1009153. doi: 10.1371/journal.pgen.1009153 (PMC7721131; doi:10.1371/journal.pgen.1009153)

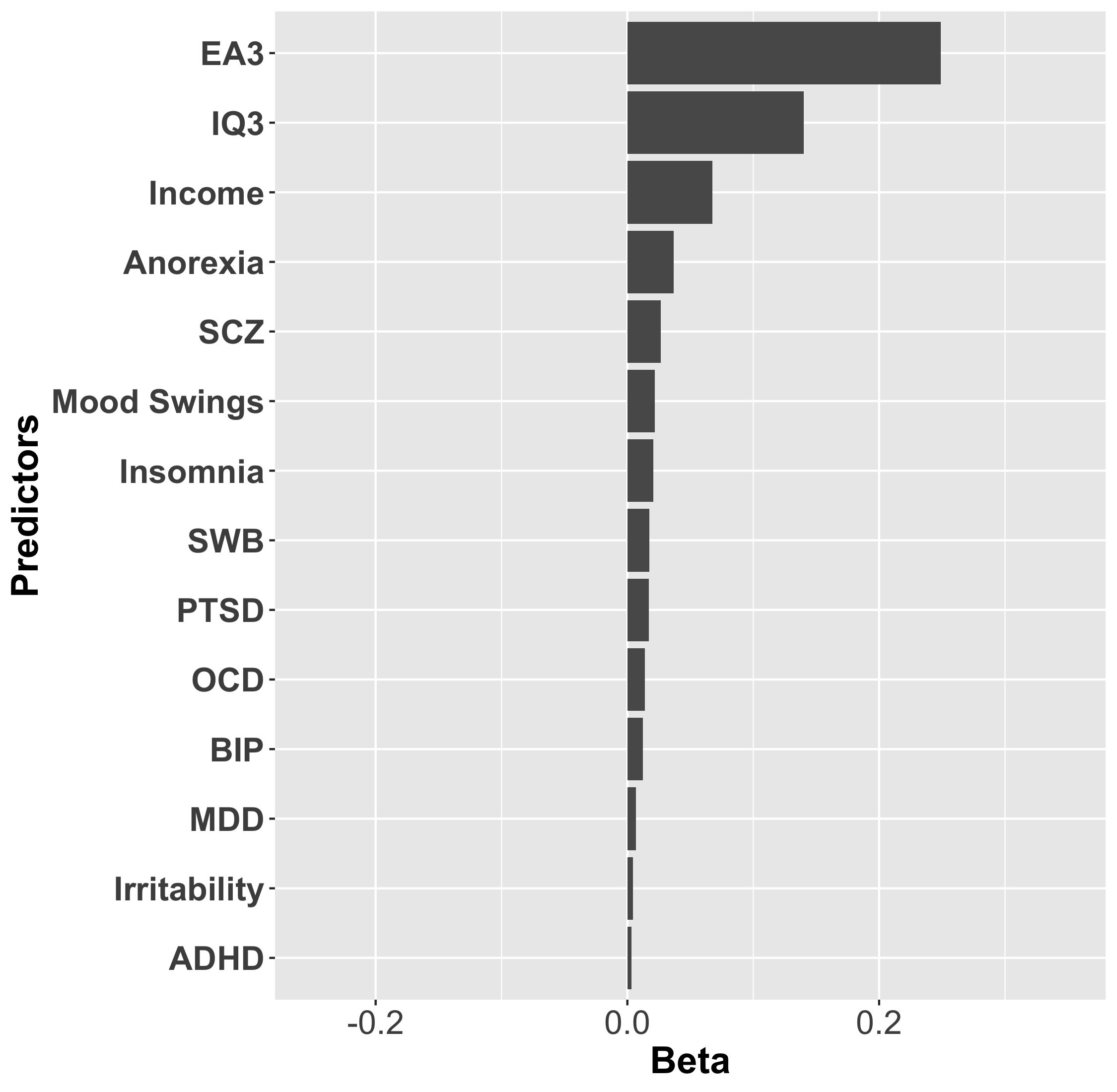

Supplement: S1 Fig — Variables importance for the best G model selected via repeated cross-validation in the training set. Note. ASD = Autism Spectrum Disorder, ADHD = Attention-Deficit Hyperactivity Disorder, BIP = Bipolar Disorder, EA3 = educational attainment, IQ3 = intelligence, MDD = Major Depressive Disorder, SWB = Subjective Well-Being, OCD = Obsessive Compulsive Disorder, PTSD = Post-Traumatic Stress Disorder, SCZ = Schizophrenia. (TIF) [file pgen.1009153.s009.tif]

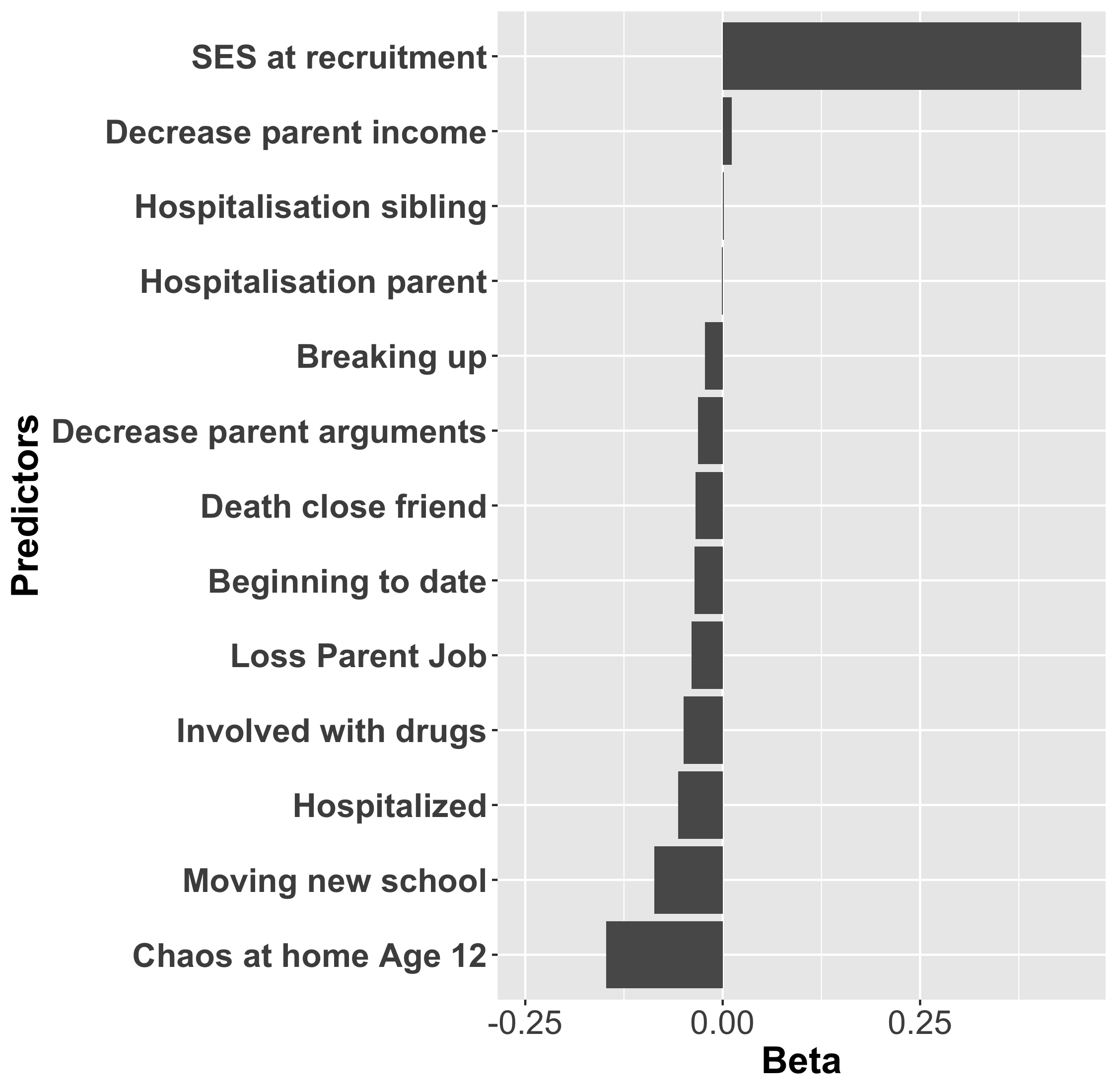

Supplement: S2 Fig — Variables importance for the best E model selected via repeated cross-validation in the training set. (TIF) [file pgen.1009153.s010.tif]

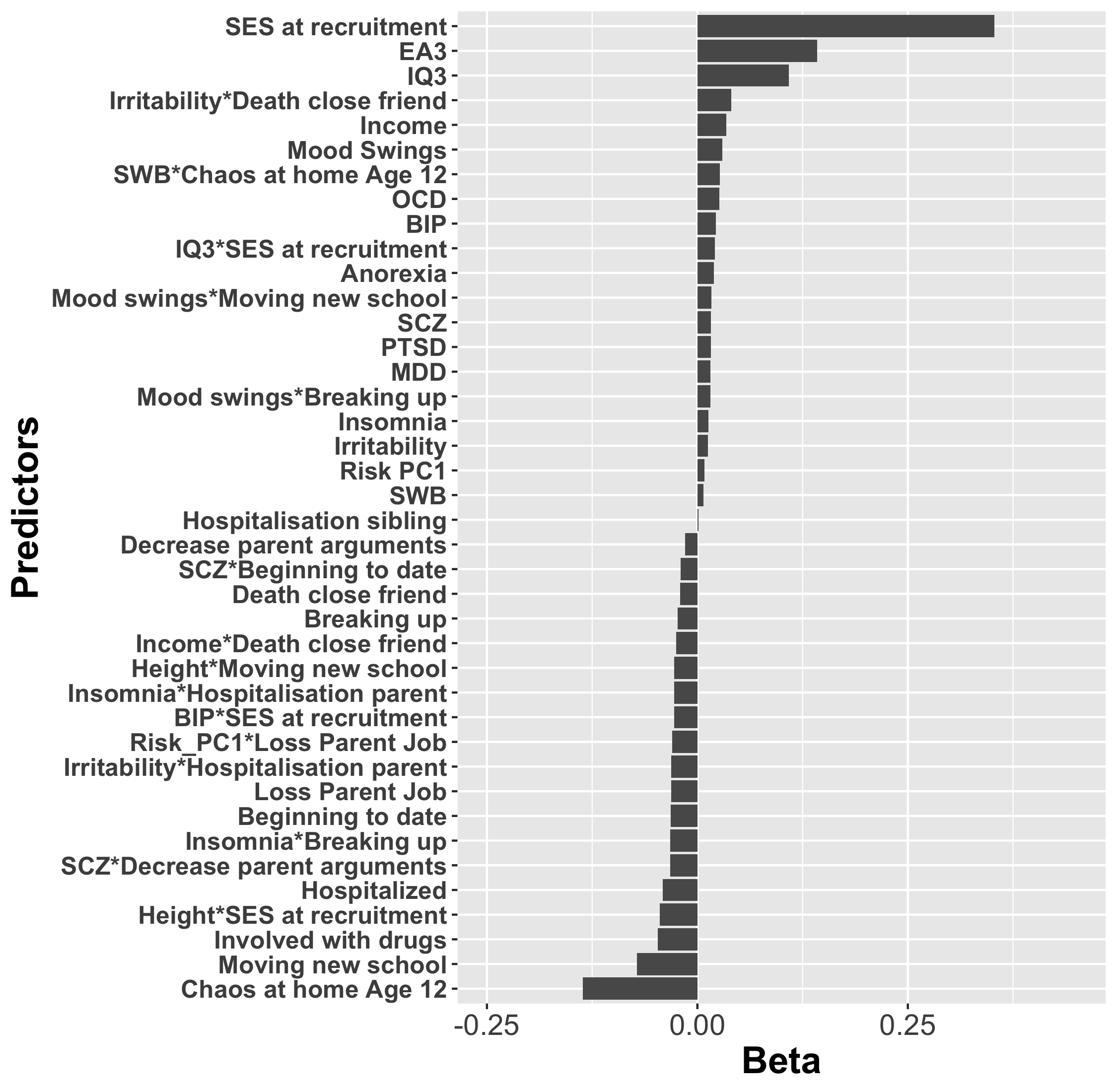

Supplement: S3 Fig — Variables importance for the best G*E model selected via repeated cross-validation in the training set. Note. For interactions the first name refers to polygenic scores, the second name refers to environmental predictors. ASD = Autism Spectrum Disorder, ADHD = Attention-Deficit Hyperactivity Disorder, BIP = Bipolar Disorder, EA3 = educational attainment, IQ3 = intelligence, MDD = Major Depressive Disorder, SWB = Subjective Well-Being, OCD = Obsessive Compulsive Disorder, PTSD = Post-Traumatic Stress Disorder, SCZ = Schizophrenia. (TIF) [file pgen.1009153.s011.tif]
