# Supplementary material for: Multivariable G-E interplay in the prediction of educational achievement
Source: PLoS Genet. 2020 Nov 17;16(11):e1009153. doi: 10.1371/journal.pgen.1009153 (PMC7721131; doi:10.1371/journal.pgen.1009153)
Supplement: Attachment — Submitted filename: RC_PGENETICS-D-20-00796.docx [file pgen.1009153.s012.docx]

Reviewer Comments

This paper investigates G-E interplay in the prediction of educational achievement (EA) with UK Twin study data. Genome-wide polygenic scores (GPS) for 20 traits and 13 environments measures that might related to EA are considered to enhance the level of understanding of G-E interplay. The penalized regression models are used to compare the prediction and model performance. Genotype-environment correlation is quantified by the correlation between the EA predicted values from the G and from E and G-E relationship is further investigated by mediation analysis. Two-way GxE effects on the other hand, tested by a hierarchical group lasso method. Overall, the paper is well written and investigates a very important question where authors try to disentangle genotype-environment correlation (rGE) and genotype-environment interaction (GxE). I think it is a good paper although I have one main concern as following.

The GPS are constructed using Lassosum method. This method is different from traditional p-value thresholding or p-value thresholding-clumping method and it is not clear if this method is robust across various traits. It is hard to understand why, for example in supplementary table S7, N of best beta changes dramatically across the traits (for some traits N is only 1 or 4 and for some traits N is more than 10,000). And the R2% in validation for some traits seems very small, for example it is only 0.0052% for ADHD and R2% varies largely among traits. I think it is good to create GPS based on traditional p-value thresholding (or thresholding +clumping to account for LD structure) and check how robust the results that authors may have.

Small comments:

1. Page 10, line 196, should be Panel D.
2. Page 11, line 210, there are two GPS after EA3, should delete one.
3. Page 13, line 243-245. The effects of G_{ea} on EA (beta=0.43; …) were reduced by 40% after introduction of the E_{eq} mediator in the model (beta=0.45; …). How beta=0.43 reduce 40% to beta=0.45?
4. Page 16 line 304 and page 17 line 309. In these two parts polygenetic scores seems have different meanings (household income for first and EA for second). It is a little bit confusing.
5. Page 21, line 405-406 and line 410-411. I am not sure how 7,026 unrelated individuals comes from a twin study, did these are individuals from one of monozygotic twins? And why dizygotic twins are not used?
6. Page 22, line 431, SES at recruitment (mean age=18 months). Here age is not clear, is the age of children or other age?
7. Page 22, line a composite of moth and father qualification … It is not clear how composite has been done and details is not given in supplementary.
