# Supplementary material for: Multivariable G-E interplay in the prediction of educational achievement
Source: PLoS Genet. 2020 Nov 17;16(11):e1009153. doi: 10.1371/journal.pgen.1009153 (PMC7721131; doi:10.1371/journal.pgen.1009153)
Supplement: Attachment — Submitted filename: Review PLOS Genetics June2020.docx [file pgen.1009153.s013.docx]

**Multivariable G-E interplay in the prediction of educational achievement**

The purpose of the paper is to investigate gene-environment correlation (rGE) and interaction (GxE) in the joint analysis of multiple genome-wide polygenic scores (GPS) and multiple environmental measures in predicting tested educational achievement (EA).

Study population: A sample of 7,026 16-year-olds, with 20 GPS for psychiatric, cognitive and anthropometric traits, and 13 environments (including life events, home environment, and SES) measured earlier in life.

Methods: First, polygenic scores were modeled (henceforth G model) and environmental measures (henceforth E model), separately and jointly (full model), to predict educational.

The relative contributions of the employed predictors to the full model were assesed.

Secondly, the authors separated direct from mediated effects of the multivariable G and E models on EA and assessed rGE defined in terms of the GPS and environmental measures employed.

Finally, GxE were assessed to systematically discover two-way interactions between all GPS and environmental measures, and test their improvement in prediction of EA.

Results:

In the G+E model EA-increasing variables included; SES in early life, GPS for educational attainment and the GPS for intelligence. EA-decreasing variable: ‘chaos at home’. The rest of the 20 GPS’s and 13 E variables were not statistically significant in the hold out model.

They found gene-environment correlation r = 0.38 (0.30 - 0.45), indicating the extent of overlapping information between the G and E models in hold out set prediction

The G+E model predicted significantly better that the E model (median R2 diff = 5.9%; 95% CI = 2.8, 9.1) and better than the G model (median R2 diff = 17.07%; CI = 13.2, 22.3).

They estimated that 40% (31%-49%) of G effects on EA were explained by environmental mediation. Vice versa, the E effects on EA were subject to a reduction of 18% (12%-25%) when accounting for the polygenic model.

No evidence were found that GxE effects significantly contributed to multivariable prediction.

It is an interesting subject, and comprehensively carried out. Multiple correlated predictors can cause multi-collinearity and overfitting, therefore penalized methods are relevant in this setting.

My main issue is the choices of predictors:

1) In the measure of SES, the authors included mother age at birth. Why not keep does two separately?
2) How was it decided which GPS’s to include. Eg. maternal age at birth would be obvious to include.
3) The chaos in home were included as a scale based on several measures of home environment, whereas life events were included separately. Why use different approaches?

Other questions to the authors:

4) Ethnicity of the sample?
5) In the introduction, it would be helpful to know a bit more about the analyses. The part I miss, is that the cross validation analysis were performed in 80% of the sample, and the subsequent hold-out analyses are in the 20% rest. Right?

Misprints?

Figure1 in the legendit says corss validation. And it say Panel C twice.

Line 368 say capture environmental influences, should that be genetic influences?
