# Supplementary material for: Multivariable G-E interplay in the prediction of educational achievement
Source: PLoS Genet. 2020 Nov 17;16(11):e1009153. doi: 10.1371/journal.pgen.1009153 (PMC7721131; doi:10.1371/journal.pgen.1009153)
Supplement: Attachment — Submitted filename: response_reviewer_comments_31_07_2020.docx [file pgen.1009153.s014.docx]

We sincerely appreciate the valuable comments and thoughtful insights from the reviewers, which substantially strengthened the manuscript. In our response, we address each of the comments in order:


**Reviewer #1:**

Reviewer Comments

This paper investigates G-E interplay in the prediction of educational achievement (EA) with UK Twin study data. Genome-wide polygenic scores (GPS) for 20 traits and 13 environments measures that might related to EA are considered to enhance the level of understanding of G-E interplay. The penalized regression models are used to compare the prediction and model performance. Genotype-environment correlation is quantified by the correlation between the EA predicted values from the G and from E and G-E relationship is further investigated by mediation analysis. Two-way GxE effects on the other hand, tested by a hierarchical group lasso method. Overall, the paper is well written and investigates a very important question where authors try to disentangle genotype-environment correlation (rGE) and genotype-environment interaction (GxE). I think it is a good paper although I have one main concern as following.

The GPS are constructed using Lassosum method. This method is different from traditional p-value thresholding or p-value thresholding-clumping method and it is not clear if this method is robust across various traits. It is hard to understand why, for example in supplementary table S7, N of best beta changes dramatically across the traits (for some traits N is only 1 or 4 and for some traits N is more than 10,000). And the R2% in validation for some traits seems very small, for example it is only 0.0052% for ADHD and R2% varies largely among traits. I think it is good to create GPS based on traditional p-value thresholding (or thresholding +clumping to account for LD structure) and check how robust the results that authors may have.

We thank the reviewer for the suggestion. We note that Lassoum has been shown by previous work to perform comparably to LDpred and to perform better than a more traditional p-value clumping and thresholding approach across diverse traits (Mak, Porsch, Choi, Zhou, & Sham, 2017; Privé, Arbel, & Vilhjálmsson, 2020). Here we employed Lassosum consistent with our previous observation that this approach seems to work better than other approaches at least for cognitive-relevant traits (Allegrini et al., 2019).

Regarding the difference that the reviewer notes between the different polygenic scores validation parameters: “N of best beta changes dramatically across the traits (for some traits N is only 1 or 4 and for some traits N is more than 10,000).” Parameter tuning can vary considerably depending on the sample used and on the particular trait. There is no guarantee (nor it would be expected) that the optimal combination of shrinkage and sparsity parameters (s and lambda) should be equivalent across scores. In this regard it is not surprising that the corresponding validation parameters (N best beta and R^2^% validation) vary depending on trait of interest, sometimes considerably.

This is akin to the optimization that can be performed with p-value thresholding (PT) in standard approaches, or priors in LDpred: we wouldn’t expect the same p-value threshold, or prior, to be the optimal parameter across different traits. For example, in LDpred, constructing a polygenic score for educational attainment with a fraction of causal markers of 1 might be optimal for prediction of educational attainment, but not for the prediction of standing height. Similarly, different polygenic scores thresholds for different traits, would not be expected to predict the same amount of variance in an outcome of interest (i.e., R^2^ will vary across thresholds and across polygenic scores).

On the other hand, regarding the very small validation R^2^% of ADHD, this could be because the ADHD polygenic score is only a weak predictor of educational achievement or that it is a particularly weak predictor in this TEDS subsample, just by random fluctuation.

To put things in context, in the hold-out set, the lassosum-based ADHD polygenic score had a more reasonable predictive power (orange bar in Figure 1 below), although still weak and not significant. To address the reviewer concern further, we tested different LDpred-based ADHD polygenic scores fractions for association with EA (in this same TEDS subset) to compare the relative predictive power of the different scores with the lassosum-based ADHD polygenic score. As shown in Figure 1 below, there was not a substantial difference between these scores and the lassosum-based score. Furthermore, in all cases the association between the different polygenic scores and EA in this particular subsample was not significant (p-values shown on top of bars).

Figure 1. Relative predictive accuracy of LDpred-based ADHD polygenic scores and lassosum-based ADHD polygenic score vs Educational Achievement (EA) in hold-out set.

Figure shows the percent of variance predicted (R^2^%) by each polygenic score (with different fractions of causal markers for LDpred, akin to optimization that can be done with p-value thresholds in a p-value clumping + thresholding approach) in EA in the hold out set, and the relative p-value for the association test on top of corresponding bars.

Small comments:

1. Page 10, line 196, should be Panel D.

This has now been changed from panel C to panel D.

2. Page 11, line 210, there are two GPS after EA3, should delete one.

This has now been amended.

3. Page 13, line 243-245. The effects of G_ea on EA (beta=0.43; …) were reduced by 40% after introduction of the E_eq mediator in the model (beta=0.45; …). How beta=0.43 reduce 40% to beta=0.45?

This was a mistake in reporting, it has now been amended to reflect the correct estimates as reported in supplementary table 4 i.e.

“…were reduced by 40% after introduction of the E_ea_ mediator in the model (*β* = 0.26; bootstrapped 95% CIs = 0.19, 0.33)”

4. Page 16 line 304 and page 17 line 309. In these two parts polygenetic scores seems have different meanings (household income for first and EA for second). It is a little bit confusing.

The legend of Figure 3 has now been amended to avoid confusion, as well as line 306 of the discussion.

5. Page 21, line 405-406 and line 410-411. I am not sure how 7,026 unrelated individuals comes from a twin study, did these are individuals from one of monozygotic twins? And why dizygotic twins are not used?

Current analyses were limited to the genotyped sample, and we retained only one individual at random from sibling pairs. We have now made this more explicit in the methods section (line 422 page 21).

6. Page 22, line 431, SES at recruitment (mean age=18 months). Here age is not clear, is the age of children or other age?

This refers to children’s age, the sentence now reads:

*“* SES at recruitment (mean children age = 18 months) was calculated…” (line 441 page 22)

7. Page 22, line a composite of moth and father qualification … It is not clear how composite has been done and details is not given in supplementary.

SES at recruitment was calculated as the mean of five standardized scores as listed in methods. The relevant section at line 441 page 22 now reads as follows:

*“*SES at recruitment (mean children age = 18 months) was calculated as the mean composite score of five standardized measures including mother and father qualification levels ranging from 1 = ‘no qualifications’ to 8 = ‘postgraduate qualification’, mother and father employment status (Surveys, 1991), and mother’s age at birth of first child’. “

**References**

Allegrini, A. G., Selzam, S., Rimfeld, K., von Stumm, S., Pingault, J.-B., & Plomin, R. (2019). Genomic prediction of cognitive traits in childhood and adolescence. *Molecular psychiatry, 24*(6), 819-827.

Mak, T. S. H., Porsch, R. M., Choi, S. W., Zhou, X., & Sham, P. C. (2017). Polygenic scores via penalized regression on summary statistics. *Genetic epidemiology, 41*(6), 469-480.

Privé, F., Arbel, J., & Vilhjálmsson, B. J. (2020). LDpred2: better, faster, stronger. *bioRxiv*.

Surveys, O. o. P. a. C. (1991). Standard occupational classification. *Her Majesty's Stationery Office volume 3*.

**Reviewer #2:**

**Multivariable G-E interplay in the prediction of educational achievement**

The purpose of the paper is to investigate gene-environment correlation (rGE) and interaction (GxE) in the joint analysis of multiple genome-wide polygenic scores (GPS) and multiple environmental measures in predicting tested educational achievement (EA).

Study population: A sample of 7,026 16-year-olds, with 20 GPS for psychiatric, cognitive and anthropometric traits, and 13 environments (including life events, home environment, and SES) measured earlier in life.

Methods: First, polygenic scores were modeled (henceforth G model) and environmental measures (henceforth E model), separately and jointly (full model), to predict educational.

The relative contributions of the employed predictors to the full model were assesed.

Secondly, the authors separated direct from mediated effects of the multivariable G and E models on EA and assessed rGE defined in terms of the GPS and environmental measures employed.

Finally, GxE were assessed to systematically discover two-way interactions between all GPS and environmental measures, and test their improvement in prediction of EA.

Results:

In the G+E model EA-increasing variables included; SES in early life, GPS for educational attainment and the GPS for intelligence. EA-decreasing variable: ‘chaos at home’. The rest of the 20 GPS’s and 13 E variables were not statistically significant in the hold out model.

They found gene-environment correlation r = 0.38 (0.30 - 0.45), indicating the extent of overlapping information between the G and E models in hold out set prediction

The G+E model predicted significantly better that the E model (median R2 diff = 5.9%; 95% CI = 2.8, 9.1) and better than the G model (median R2 diff = 17.07%; CI = 13.2, 22.3).

They estimated that 40% (31%-49%) of G effects on EA were explained by environmental mediation. Vice versa, the E effects on EA were subject to a reduction of 18% (12%-25%) when accounting for the polygenic model.

No evidence were found that GxE effects significantly contributed to multivariable prediction.

It is an interesting subject, and comprehensively carried out. Multiple correlated predictors can cause multi-collinearity and overfitting, therefore penalized methods are relevant in this setting.

My main issue is the choices of predictors:

1) In the measure of SES, the authors included mother age at birth. Why not keep does two separately?

While it might be interesting to test mother age at birth separately, here we were interested in the construct of socio-economic status (SES). This measure was constructed as the mean composite score of five standardized measures, including mother and father qualification levels ranging from 1 = ‘no qualifications’ to 8 = ‘postgraduate qualification’; mother and father employment status; and mother’s age at birth of first child has been previously used in TEDS, which is robustly associated with different SES measures later in life (Krapohl & Plomin, 2016).

2) How was it decided which GPS’s to include. Eg. maternal age at birth would be obvious to include.

Looking at the effects of the polygenic score for maternal age at birth might be an interesting avenue for future research following up on this study, however as noted in the discussion section several other predictors might be employed both at the level of measured environments and polygenic cores. Here we limited the analyses to the most powerful and publicly available polygenic scores for cognitive, psychopathology and anthropometric related traits, as stated in the limitation section of the discussion.

We now clarify this also in the measures section at line 494:

“We employed the most powerful and publicly available GWAS summary statistics for cognitive, psychopathology and anthropometric traits.”

3) The chaos in home were included as a scale based on several measures of home environment, whereas life events were included separately. Why use different approaches?

The Chaos at home measure is a validated composite that we have employed in many other studies previously (e.g. Hanscombe, Haworth, Davis, Jaffee, & Plomin, 2010). While it might be interesting to separate each component of this scale, we note that the correlations among the Chaos at home items are very high, which is why we chose to analyse them as a unitary construct of (chaotic) home environment.

Life events, on the other hand, are better suited to be analysed separately since these often represent stochastic events unrelated to each other, each with potentially very different effects on EA.

Other questions to the authors:

4) Ethnicity of the sample?

This is specified in the supplementary material, we only retained people of European ancestry. For clarity we have now also specified this in the sample section in the main body of the manuscript.

Line 416 now reads

”including 3,320 dizygotic twin pairs and 7,026 unrelated individuals, all of European ancestry”

6) In the introduction, it would be helpful to know a bit more about the analyses. The part I miss, is that the cross validation analysis were performed in 80% of the sample, and the subsequent hold-out analyses are in the 20% rest. Right?

Yes, this is reported in the methods section, but not in the introduction. We have now specified this at line 159 of the introduction:

“Models were tuned using repeated cross-validation in 80% of the sample and tested in the remaining 20% hold-out set.”

Misprints?

Figure1 in the legendit says corss validation. And it say Panel C twice.

These have been amended.

Line 368 say capture environmental influences, should that be genetic influences?

Here we mean that there might be other variables that can be argued to represent environmental effects relevant to educational achievement. To clarify line 373 now reads:

“however, many other variables can be argued to capture EA relevant environmental influences”.

**References**

Hanscombe, K. B., Haworth, C. M., Davis, O. S., Jaffee, S. R., & Plomin, R. (2010). The nature (and nurture) of children's perceptions of family chaos. *Learning and individual differences, 20*(5), 549-553.

Krapohl, E., & Plomin, R. (2016). Genetic link between family socioeconomic status and children’s educational achievement estimated from genome-wide SNPs. *Molecular psychiatry, 21*(3), 437-443.

**Reviewer #3:** In this paper, the authors consider the joint role of genome-wide polygenic scores (GPSs) and environmental variables at predicting educational achievement. While it's known that such measures are known to be individually good predictors of educational variables, this study quantifies the degree of overlapping signal between a set of genetic and environmental variables. At a high level, they do this using regularized prediction models and mediation-style analyses. They find that 40% of the predictive power of GPSs and 18% of the predictive power of environmental variables is due to the correlation between these variables. They also test whether interactions of environmental and genetic variables add to the predictive power of joint genetic-environmental predictive models, and find no evidence that interactions of the variables they considered contribute significantly to multivariable prediction.

This paper represents an impressive effort in a rich data set. There is a lot of interesting information presented. Also the paper was easy to read, the analyses were clearly described in a way that I believe I would be able to replicate the analyses, and the tables and figures had clear and comprehensive captions. That said, upon reading the paper, I found myself with a lot of questions about what the major take-aways are for this project.

Major concerns:

1) How does this paper fit relative to other papers that do similar work? The authors make several strong statements about the novelty of their work, but there are a number of papers that ask very closely related questions. For example, Selzam et al (2019) compare the within- and between-family predictive power of GPSs for educational achievement, which would represent the passive rGE that the authors describe. Also, Lee et al. (2018) contains an analysis quite similar to this one looking at the incremental R2 of an education GPS at predicting educational attainment above several environmental variables. While educational attainment and educational achievement are different variables, they are very highly genetically correlated. It would be helpful if the authors could explicitly highlight what we are learning from this paper relative to related existing work.

We agree with the reviewer that some of our results provide converging evidence with previous work. However, we note that there are substantial differences between our analyses and conclusions that can be drawn from these previous works.

1. First, multivariable vs univariate framework: in the Selzam et al., 2019 paper only univariate analyses were carried out. The scores were not considered conditional on other polygenic scores, let alone with related measured environments.

Furthermore, while the GE correlation focus is common to both papers, in the present study we mainly focus on the GE interplay (both interaction and correlation) impact on prediction of educational achievement. As such our paper provides a number of different, additional, analytical layers compared to the Selzam paper.

2. Second, focus on both polygenic scores and measured environments: In the present study we consider the relative effects of both measured environmental and genomic predictors on EA. As mentioned, the focus of the Selzam et al., 2019 paper, as well as the analyses carried out in the Lee et al., 2018 paper were mainly on univariate polygenic scores effects. This enabled us to test the relative percent decrease in EA-relevant environmental prediction effects that is due to polygenic scores effects as well as the other way around.
3. Third, statistical inference of trait associations: for a similar reason, while we agree that the Lee et al., 2018 paper provides a similar analysis to the one we show in figure 1b and 1d of the manuscript, we highlight that our analysis has several conceptual differences with the Lee et al. analysis.
   a. Firstly, we compared the relative difference between models including multiple environmental measures, multiple polygenic scores, a combination of both, and their two-way interactions.
   b. Secondly, the actual environmental measures employed are more ‘fine grained’ and proximal than the analyses carried out by Lee et al., 2018 in that we also include measures of the home environment and of life events.
   c. Thirdly, we take this a step further by formal statistical inference of trait associations of the relative polygenic scores and environmental measures when jointly considered in the same model. This latter part alone allowed us to make novel observations that were not possible in the Lee et al. analyses, for example that both polygenic scores for IQ and EA are significant within the same model conditional on each other, other polygenic scores and relevant environmental measures such as SES and chaos at home.

We believe that these points differentiate substantially our results and those that can be drawn thereof from either the Selzam et al., 2019 or Lee et al., 2018 papers.

We agree with the reviewer that the manuscript would benefit from explicitly comparing our analyses and those of previous studies. We have added the following paragraph in the Discussion:

“Previous work (Lee et al., 2018) showed that the predictive accuracy of EA3 provides unique information beyond correlated demographics and distal control variables such as income and parental educational attainment. Here we extend this observation to multiple polygenic scores within a prediction framework, as well as multiple measured environments, including proximal measures of home environment and life events. Furthermore, we take this a step further by formally testing trait associations of the relative polygenic scores and environmental measures when jointly considered in the same model.”

We also revised the paragraph about the Selzam et al. study as follows:

“Previous work (Selzam et al., 2019) has shown that prediction of educational achievement by EA3 GPS consistently decreases within-family, suggesting that passive gene-environment correlation explains part of the predictive power of EA3. Here we show that reciprocal indirect effects between multivariable E and G models explain a substantial proportion of variation of their direct effects on EA. These results provide converging evidence with recent research looking at rGE underlying parenting and children educational attainment (27, 47).”

2) I had a difficult time interpreting their rGE results for a number of reasons.

a) How does the sparse variable selection procedure affect the authors' estimates? As far as I could tell, the authors selected their genetic and environmental variables using an elastic net procedure in the same step. If there is strong rGE (as the authors find) some of the genetic variables that are highly correlated with the environmental variables may be selected in the place of the environmental variables in the penalization process, even if they represent independent signals. Likewise, some environmental variables will be selected in the place of the genetic variables. Since you are removing variables that are correlated with each other, this would have an effect on the subsequent rGE analysis. It may be possible to address this concern by selecting the genetic and environmental variables separately.

We note that elastic net tends to retain groups of correlated predictors instead of discarding one at random (behaviour more typical of the Lasso method) overcoming the problem highlighted by the reviewer. In fact, we note that top variables selected by the G model and E model independently are included in the full GE model.

Furthermore, this would not have a bearing on subsequent rGE analyses because while we jointly considered polygenic scores and environmental predictors in the full model (Figure 1 panel C, and Figure 1 colour green in panel A and panel B), we only employed the separate polygenic scores and environmental models (blue and orange colours in panel A and panel B of figure 1) for mediation analyses (Figure 4) as well as to test the correlation between the two (page 28, line 574).

b) How appropriate is it to estimate the mediation analysis in both directions? In order to interpret the estimates of how much the environmental variables mediate the genetic variables, you need to assume the model in Figure 4B. To estimate the amount of genetic confounding, you need to assume the model described in line 324 of the manuscript. Since both of these models can't be simultaneously true, I believe it can't be the case that the two estimates in lines 35 to 39 are both true.

We agree with the reviewer that both models cannot be true at the same time. In fact, only model 4b is plausible, where environmental effects mediate polygenic scores effects. Model 4c is rather a statistical abstraction as we note at line 598 of the methods section, and imply at page 17 line 323 in the discussion. This model is fit only to statistically quantify the amount of genetic ‘confounding’ described in line 325. In fact, as mentioned at line 633 of the methods, mediation and confounding effects are statistically equivalent (but they are conceptually distinct), and confounding effects can be estimated with mediation (MacKinnon, Krull, & Lockwood, 2000; Pingault et al., 2019). In this sense we are not implying that both models are true at the same time, but just that polygenic scores effects partly capture environmental effects and are therefore attenuated when we take this into account. Similarly, environmental effects will be partly biased upwards due to genetic confounding.

We now added a clarification in text at line 601 of the methods:

“In other words, confounding and mediation effects are statistically equivalent, such that they can both be estimated by mediation analysis, but conceptually distinct (77).”

c) How do errors in variables affect the interpretation of these analyses? For example, the 'chaos at home' variable is likely a noisy proxy for the true amount of chaos at home. This means that even if 100% of the genetic signal were mediated through this variable, the GPS would still remain predictive after including that variable in the regression. Similarly, the polygenic scores may be thought of as noisy measures of the true additive genetic component. If that's the case, then a mediation analysis will overestimate the amount that the environmental variables mediate the predictive power of the genetic factor.

We agree with the reviewer that measures employed, environmental as well as polygenic scores, are just noisy proxies of the true phenomena that they represent. However, we note that this can be said for any other measure. We agree that insofar as these are not perfect proxies then we would have a degree of over (or under) estimation of environmentally mediated effects or genetic confounding. Our multivariable approach partly overcomes this issue in that considering multiple scores jointly we are less concerned about the extent to which a single variable is a proxy for its true measurement, but rather whether globally they are proxy for EA-relevant environmental effects or polygenic predisposition.

Notwithstanding this we agree with the reviewer that this is a problem that warrants consideration, therefore we added a cautionary note for the reader at page 20 line 383 of the discussion:

“We note that the environmental variables employed in the models jointly represent only a noisy proxy for the true EA relevant environmental effects, just as the multi-polygenic scores model is a noisy proxy for the true additive genetic predisposition to EA. As such, estimates derived from mediation analyses will imprecisely capture the extent to which E and G models are reciprocally mediated.”

Minor concern:

3) The notation used the methods section is confusing. The authors often use the same variables to represent different things. For example, in the equation on line 463, the authors use 'r' to denote both a vector covariances and also as an indicator that the genotypes come from a reference sample. Also the variables and coefficients in line 463 correspond to different variables and parameters in line 505 despite having the same names.

For consistency we used the original notation from the papers where the equations were taken, followed by a detailed explanation of what the relevant indices mean. We agree with the reviewer however that this might be confusing for the reader and we harmonized the notations as follows:

The notation at page 24, line 473 onwards, has been changed from X_r_ to X_ref_, while at page 25, line 517 onwards, X has been changed to X’.

**References**

MacKinnon, D. P., Krull, J. L., & Lockwood, C. M. (2000). Equivalence of the mediation, confounding and suppression effect. *Prevention science, 1*(4), 173-181.

Pingault, J.-B., Rijsdijk, F., Schoeler, T., Choi, S. W., Selzam, S., Krapohl, E., . . . Dudbridge, F. (2019). Estimating the sensitivity of associations between risk factors and outcomes to shared genetic effects. *bioRxiv*, 592352.

Selzam, S., Ritchie, S. J., Pingault, J. B., Reynolds, C. A., O’Reilly, P. F., & Plomin, R. (2019). Comparing within-and between-family polygenic score prediction. The American Journal of Human Genetics, 105(2), 351-363.

Lee, J. J., Wedow, R., Okbay, A., Kong, E., Maghzian, O., Zacher, M., ... & Fontana, M. A. (2018). Gene discovery and polygenic prediction from a 1.1-million-person GWAS of educational attainment. Nature Genetics, 50(8), 1112.
